# Mental Disorders and Level of Resilience in Eight High-Altitude Cities of Peru during the Second Pandemic Wave: A Multicenter Population-Based Study

**DOI:** 10.3390/ijerph20010519

**Published:** 2022-12-28

**Authors:** J. Pierre Zila-Velasque, Pamela Grados-Espinoza, Naomi Coba-Villan, Jocelyn Quispe-Chamorro, Yesenia F. Taipe-Guillén, Estefany Pacheco, Laura Ccasa-Valero, Virgilio E. Failoc-Rojas, Cristian Díaz-Vélez, Mario J. Valladares-Garrido

**Affiliations:** 1 Facultad de Medicina Humana, Universidad Nacional Daniel Alcides Carrión, Pasco 19001, Peru; 2 Red Latinoamericana de Medicina en la Altitud e Investigación (REDLAMAI), Pasco 19001, Peru; 3 Facultad de Medicina Humana, Universidad Nacional de Cajamarca, Cajamarca 06001, Peru; 4 Facultad de Medicina Humana, Universidad Nacional del Centro del Peru, Junin 12007, Peru; 5 Escuela Profesional de Medicina Humana, Universidad Nacional San Cristóbal de Huamanga, Ayacucho 05003, Peru; 6 Escuela de Medicina Humana, Universidad Continental, Junin 12000, Peru; 7 Facultad de Medicina Humana, Universidad Nacional del Altiplano, Puno 21001, Peru; 8 Unidad de Generación y Síntesis de Evidencia, Universidad San Ignacio de Loyola, Lima 15024, Peru; 9 School of Medicine, Universidad Privada Antenor Orrego, Trujillo 13008, Peru; 10 Oficina de Inteligencia Sanitaria, Hospital Nacional Almanzor Aguinaga Asenjo, EsSalud, Chiclayo 14001, Peru; 11 South American Center for Education and Research in Public Health, Universidad Norbert Wiener, Lima 15046, Peru; 12 Oficina de Epidemiología, Hospital Regional Lambayeque, Chiclayo 14012, Peru

**Keywords:** COVID-19, mental health, resilience, public health, altitude

## Abstract

COVID-19 has led us to take preventive measures, such as social isolation, to reduce the high transmissibility of the disease. This could have affected the mental health of various population groups and the development of resilience as a mitigator. A cross-sectional analytical study was conducted with 700 participants from eight cities. The dependent variables were depression, anxiety, and post-traumatic stress disorder (PTSD). The independent variable was resilience. Generalized logistic regressions were used to identify the associations between the variables. The population consisted mostly of university students (65.0%); the rest of the population was distributed among workers of public or private institutions, housewives, and others (35.0%). High prevalences of anxiety (72.7%), depression (64.1%), and PTSD (15.1%) were found, as well as a median (interquartile range) resilience score of 24 points was determined. Factors associated with a high prevalence of PTSD were having lost employment and having a family member who died from COVID-19. For depression, associated factors were severe food insecurity and hypersomnia. For anxiety, associated factors were were having a deceased family member with COVID-19 and mild food insecurity. Our results show that, during the pandemic, the general population had a higher prevalence of mental disorders. In addition, anxiety was the most prevalent of the dependent variables. Special attention should be paid to the factors influencing the development of mental disorders and mental health prevention and promotion programs should be established.

## 1. Introduction

In December 2019, the presence of a coronavirus that caused a severe acute respiratory syndrome (SARS-CoV-2) was detected, for the first time, in Wuhan, a city in China. On 31 January 2020, the World Health Organization (WHO) declared COVID-19 a worldwide health emergency [1]. To prevent and reduce contagion, Peru applied preventive measures proposed by the WHO. Among them, on 15 March 2020, the government decreed mandatory social isolation [2], also called a quarantine, which is the separation and restriction of the movement of people [3]. These measures, together with the fear of the COVID-19 pandemic, economic and labor problems, and the alteration of people’s daily life routines and activities, had relevant repercussions for mental health [2,4]. 

There are existing studies on the effects of the COVID-19 pandemic on the mental health of general populations, where stress, anxiety, depression, insomnia, and fear were found [5,6]. One of the first studies that assessed the impact on mental health was conducted in China, where more than 50.0% of respondents reported severe psychological impact, while 16.5% and 28.8%, respectively, reported moderate and severe depressive and anxious symptoms [7]. Likewise, another study found that mean post-traumatic stress disorder (PTSD) scores were up to four times higher for individuals who had been quarantined than for individuals who were not quarantined [5]. In this context, studies indicated that resilience would confer a protective effect with respect to the mental disorders mentioned above and represents an effective psychological coping strategy in the face of the constant threat posed by this pandemic [5].

On the other hand, mental health in Peru was already a major challenge for the authorities before the pandemic, and it constituted a public health problem. It was reflected in the various reports that found a prevalence of 17.0% of depressive episodes, 3.0% of generalized anxiety disorder. and 5.0% of post-traumatic stress disorder. In addition, in the urban areas of the Andes, the jungle, and border regions, a prevalence of mental disorders of any kind, ranging from 34.7% to 39.3% [8,9], was found. 

The pandemic could further aggravate this situation, as Peru has been classified as one of the countries with the worst response to COVID-19. Furthermore, seroprevalence values of 29.5% and 70.0% have been recorded in Lambayeque and Iquitos, respectively [9,10], and COVID-19 seropositivity of 13.8% has even been recorded at a hospital in a high Andean area [11,12]. In view of the above, our research aimed to study the association between resilience and mental disorders in eight cities in Peru at 1500 m above sea level (m.a.s.l.) during the second pandemic wave due to COVID-19.

## 2. Materials and Methods

### 2.1. Sample and Procedure

#### 2.1.1. Study Design and Population

**Figure 1:**
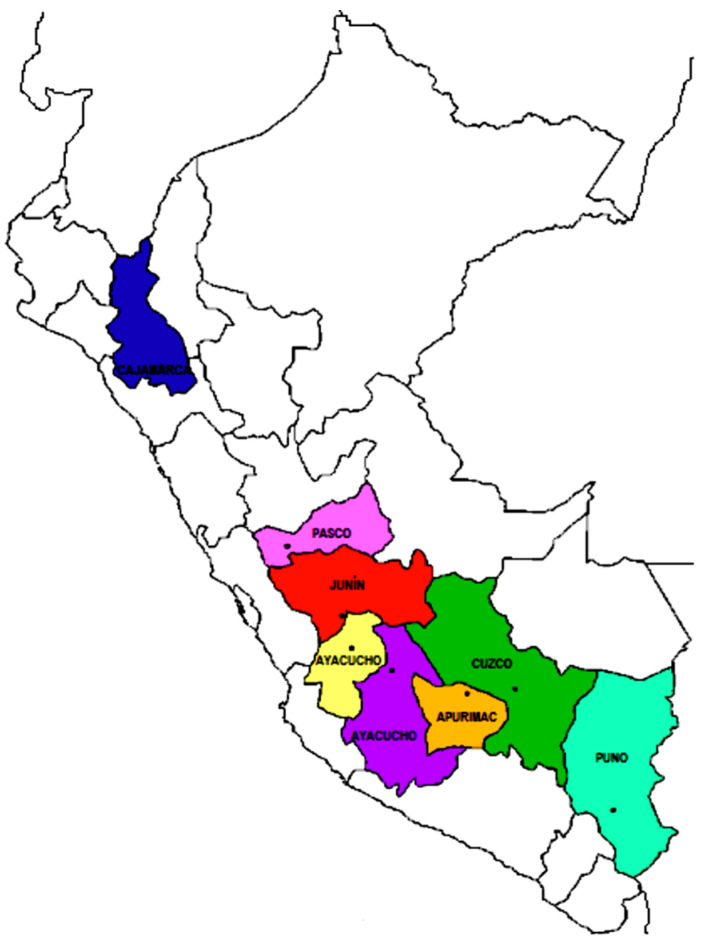
Graph of the eight cities located at more than 1500 m.a.s.l., which were the places of residence of the study participants.

We conducted an analytical cross-sectional study that identified the association between resilience and mental health disorders due to COVID-19 in eight high-altitude cities. These were cities in Peru that were higher than 1500 m.a.s.l., because this level is considered to be the limit for the development of physiological modifications [13]. The cities were Apurímac, Ayacucho, Cajamarca, Cuzco, Huancavelica, Junín, Pasco, and Puno. (Figure 1). In addition, because it was known that the higher the altitude, the lower the incidence of COVID-19 that was reported, and that high altitude behaved as a protective factor, the degree of concern and the development of mental health disorders would be less [14].

**Figure 2:**
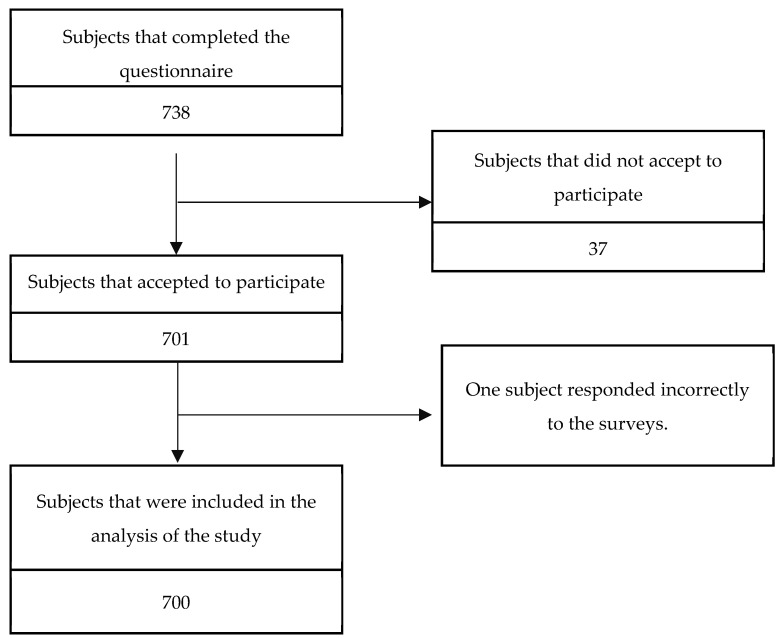
Flowchart of the selection of study participants.

Participants who were over 18 years of age and residing in the cities mentioned above were included. A total of 738 responses were obtained (Figure 2).

#### 2.1.2. Procedure

A virtual survey was developed using the Google forms platform, which was available from 20 December 2020 to 28 February 2021. The survey was disseminated mainly through social networks (WhatsApp, Instagram, Facebook, and Telegram) and institutional emails of the authors and collaborators. They disseminated the survey’s instruments within their departments, requesting the completion of the survey.

#### 2.1.3. Questionnaire

The questionnaire consisted of six sections covering (1) sociodemographic data; (2) the Generalized Anxiety Disorder Scale (GAD-7); (3) the Depression Scale (Patient Health Questionnaire, PHQ-9); (4) a sleep quality questionnaire (COS); (5) the Connor–Davidson Resilience Scale (CD-RISC); and (6) a post-traumatic stress disorder questionnaire (PCL-C).

In the general data, we obtained information on age, gender, marital status, religion, previous pathologies, educational level, self-perception of health, and time at home.

### 2.2. Measures

#### 2.2.1. Dependent Variables

Anxiety: We used the GAD-7 questionnaire, which is a unidimensional self-administered scale designed to assess the presence of generalized anxiety disorder (GAD) symptoms [15]. A cut-off point was identified, which optimized sensitivity (89%) and specificity (82%) [16]. The instrument consists of seven items with scores ranging from zero (not at all) and three (almost every day). Thus, the total score ranges from zero to 21, with higher scores indicating higher severity of anxiety. Reliability (internal consistency) was high; Cronbach’s alpha = 0.875 [17].

Depression: We used the PHQ-9 depression scale, which is a psychometrically reliable instrument for the diagnosis of depression. It is easy to use in the context of the primary care system in Peru [18]. It consists of nine items that evaluate the presence of depressive symptoms (corresponding to DSM-IV criteria) in the past two weeks. Each item has a severity index corresponding to 0 = “never”, 1 = “some days”, 2 = “more than half the days”, and 3 = “nearly every day”. The overall score ranges from 0 to 27, with higher scores indicating higher severity of depression. It shows an acceptable internal consistency with a Cronbach’s Alpha coefficient of 0.835; in addition, optimal sensitivity (88%) and specificity (92%) values [19] were found.

Post-traumatic stress disorder: We used the PCL-C instrument, which includes 17 items that correspond to the set of symptoms identified in the DSM-IV-TR for criteria B, C, and D (intrusive re-experiencing, avoidance, and hyperarousal, respectively). In the instructions, the respondents were asked how much they had been bothered by each of the 17 symptoms during the past month, using a Likert scale, where 1 meant “not at all”, 2 “a little bit”, 3 “moderately”, 4 “quite a bit”, and 5 “extremely”. The minimum total score of the instrument was 17 and the maximum score was 85, with higher scores indicarting higher severity of PTSD. According to the original version, a score equal to or higher than 44 indicates the presence of PTSD symptoms or a “possible case” [20]. The instrument showed a high level of internal consistency (α = 0.94) and adequate test-retest reliability (r = 0.82) [21].

#### 2.2.2. Main Independent Variable

Resilience: The abbreviated version of the Connor–Davidson resilience scale (CD-RISC) was used to evaluate resilience. It consists of ten items that can be used as a reliable and valid instrument. The original version has good properties: a Cronbach’s alpha of 0.89 (general population) and a test-retest reliability of 0.87 (people with generalized anxiety disorder (GAD) and post-traumatic stress disorder (PTSD)) [22]. Resilience was evaluated through a Likert scale with five optional responses, with scores ranging from zero to four. The higher the score, the higher the resilience. In general, the instrument shows excellent psychometric properties and allows an efficient measurement of resilience [23]. 

#### 2.2.3. Secondary Independent Variables

Sleep Quality (COS): This questionnaire is self-administered and helps in diagnosing sleep disorders, such as insomnia and hypersomnia, according to the DSM-IV and ICD-10 criteria. It consists of 15 items, 13 of which are grouped into three scales: subjective sleep satisfaction, insomnia, and hypersomnia. The scoring range is from 9 to 45 (the higher the score, the greater the severity of sleep disturbance). The internal consistency regarding the items comprising the insomnia scale was 0.91, while the internal consistency regarding the items comprising the hypersomnia scale was 0.88. The level of internal consistency for the total COS was 0.90. All questions were answered with a Likert-type scale. The score for the subscale of subjective sleep satisfaction ranged from one to seven points; for the subscale of insomnia, it ranged from 9 to 45 points; and for the subscale of hypersomnia, it ranged from 3 to 15 points [24].

Household Food Security Access Scale (HFIAS): This scale was developed by the United States Agency for International Development. It includes nine items that correspond to questions about food in the past four weeks. In the instrument’s instructions, the respondent is asked if the household in which he/she lives suffers from food insecurity in each period, together with the anxiety he/she may experience, the quality and insufficient intake of food, and the physical consequences. The overall score is calculated as the sum of the item scores, with higher scores indicating higher food insecurity. Responses about food insecurity are categorized as the following: food security (question 1); mild food insecurity (questions 2–4); moderate food insecurity (questions 5 or 6); and severe food insecurity (questions 6–9) [25]. The instrument showed high internal consistency (α = 0.74) [26].

### 2.3. Analysis Plan

We used descriptive statistics to examine respondents’ characteristics and responses using frequencies and percentages. We described categorical variables as frequencies and percentages, and continuous variables as mean (standard deviation) or median (range) values, as appropriate. 

A chi-square test was used to determine the associations of the variables, according to groups or categories. We performed the Mann–Whitney U test to identify differences between two groups of continuous variables. For simple and multiple regression analysis, we used generalized linear models (GLMs) with a Poisson distribution family, robust variance, a log link function, and clustering by place of residence. Prevalence ratios (PR) and 95% confidence intervals (95% CI) were estimated. Simple regression models were used to analyze the outcome association with each individual exposure. Then, all variables analyzed in the simple regression models were included in the multiple regression model. This allowed the observation of changes in the PR and 95% CI of the covariates that may act as confounders. Anxiety and depression variables were recategorized into dichotomous values (no, yes) to provide practical information for public health officials.

Survey data were organized through Microsoft Windows Excel ^®^ (licensed for computer use) and analyzed in Stata 16.1 (College Station, TX, USA: StataCorp LL).

## 3. Results

### 3.1. General Description of the Sample

The median age of the participants was 23 years, and the age range was from 18 to 70 years. More than half of the participants were women (56.7%, *n* = 394). Regarding clinical history, 15.0% (*n* = 105) of the participants reported having some comorbidity and 12.7% (*n* = 89) reported having or having had a diagnosis of COVID-19. Regarding information on biosecurity measures against COVID-19, 84.9% (*n* = 594) of the participants reported having complied with containment measures (confinement and/or isolation and/or social distancing) and 44.6% (*n* = 312) perceived the severity of the pandemic as serious. (Table 1).

### 3.2. Prevalence of Post-Traumatic Stress Disorder (PTSD), Depression, and Anxiety

Table 1 shows the results of prevalence of mental health outcomes. The prevalence of PTSD was 15.1% (*n* = 106; 95% CI: 12.57–18.02%), while the prevalence of anxiety and depression were 72.7% (*n* = 509; 95% CI: 69.25–75.98%) and 64.1% (*n* = 449; 95% CI: 60.46–67.70%), respectively.

The bivariate analysis showed significant differences in the prevalence of PTSD, according to young age (*p* = 0.005), perception of poor/very poor health (*p* < 0.001), insomnia (*p* < 0.001), and hypersomnia (*p* < 0.001). Regarding depression, significant differences were found in the following variables: young age (*p* < 0.001), female gender (*p* = 0.003), perception of fair health (*p* < 0.001), insomnia (*p* < 0.001), and hypersomnia (*p* < 0.001). Regarding anxiety, the associated factors were young age (*p* = 0.012), regular health perception (*p* < 0.001), insomnia (*p* < 0.001), and hypersomnia (*p* < 0.001) (Table 2).

### 3.3. Resilience and Other Factors Associated with Post-Traumatic Stress Disorder, Depression, and Anxiety

The multiple regression analysis showed that resilience was associated with a higher level of prevalence of PTSD (PR: 1.02; 95% CI: 1.00–1.03). In addition, the prevalence of PTSD was higher in people who had had a family member who died from COVID-19 (PR: 1.34; 95% CI: 1.02–1.74), had lost their job during the health emergency (PR: 2.21; 95% CI: 1.37–3.56), had moderate food insecurity (PR: 1.37; 95% CI: 1.05–1.78), and hypersomnia (PR: 1.27; 95% CI: 1.20–1.35). On the other hand, currently working factor reduced the prevalence of PTSD (PR: 0.53; 95% CI: 0.28–0.99) (Table 3). The log pseudolikelihood of the model was equal to −236.5.

For depression, no association with resilience was observed. People who did not respect COVID-19 containment measures (PR: 1.20; 95% CI: 1.08–1.34), had family members who had died from COVID-19 (PR: 1.19; 95% CI: 1.08–1.31), had lost their jobs (PR: 1.10; 95% CI: 1.03–1.18), had mild (PR: 1.20; 95% CI: 1.10–1.30) and severe (PR: 1.17; 95% CI: 1.10–1.25) food insecurity, and had hypersomnia (PR: 1.09; 95% CI: 1.07–1.11) had a higher prevalence of depression. On the other hand, having completed secondary education or having ongoing secondary studies decreased the prevalence of depression by 10.0% (PR: 0.91; 95% CI: 0.84–0.98) (Table 4). The log pseudolikelihood of the model was equal to −608.7.

Regarding anxiety, no association with resilience was observed. Female gender (PR: 1.15; 95% CI: 1.03–1.28), having a family member deceased due to COVID-19 (PR: 1.10, 95% CI: 1.02–1.19), having mild food insecurity (PR: 1.17, 95% CI: 1.06–1.30), and hypersomnia (PR: 1.07; 95% CI: 1.05–1.08) were associated with a higher prevalence of anxiety (Table 4). The log pseudolikelihood of the model was equal to −647.4.

## 4. Discussion

### 4.1. Prevalence of Mental Health Outcomes

In our investigation, we found that one out of ten participants had post-traumatic stress disorder due to the COVID-19 pandemic (PR: 15.1%; 95% CI: 12.57–18.02%). These results are consistent with systematic reviews that found, respectively, that PTSD symptoms were present in 15.0% and 19.3% in the general population [27,28]. Similarly, another review found that approximately one out of ten people in the general population experienced PTSD symptoms [29]. There is a similar situation in Ecuador, where the prevalence is 14.2% [30]. However, these results differ from studies conducted in China, where the prevalence reported was 7.0% [31], and from another study that reported a prevalence of 31.8% [32], a situation that was similar to the 49.0% found in a systematic review [33]. It should be noted that the included studies had health care workers and university students as a large proportion of the population. The prevalence of PTSD found in our study could be attributed to the psychological distress in the evaluated population caused by the pandemic [28,34].

In addition, we found that six out of ten participants presented with depression (PR: 64.1%; 95% CI: 60.46–67.70%). This finding differs from most studies, including systematic reviews in which the reports ranged from 15.9% to 31.4% [35,36,37,38]. However, there are different situations in Latin American countries such as Ecuador, where the prevalence was 17.7% [30], Argentina (9.5%), Chile (3.0%), Uruguay (3.0%) [39], Brazil (21.5%) [39], Venezuela (21.3%), and Mexico (38.9%) [40]. The higher prevalence found in our study may have been caused by our population being mostly young people; as is known, depression usually begins at an early age [41], and it is a common problem in this age group [42]. Furthermore, the higher prevalence may be due to rates progressing according to epidemiological waves [43], as our investigation was conducted in the context of the second pandemic wave. This is supported by the fact that the prevalence of depression may increase and be up to three times higher when compared with pre-pandemic figures [35,38], which would also explain the high prevalence in our study.

In our study, it was also found that seven out of ten people interviewed presented with anxiety (PR: 72.7%; 95% CI: 69.25–75%). This report is higher than that found in other studies that evaluated young people and university students during the COVID-19 pandemic. A cross-sectional study conducted worldwide (South Africa, Italy, USA, and Australia) reported 59.0% [44]. Other studies conducted in the Czech Republic and Slovakia reported 14.1% and 11.6%, respectively [45]; in the U.S., 51.9% [46]; in China, 11.0% [47]; and in France, 27.5% [48]. However, different results were also found in Latin America: Brazil, 47.3% [49]; Ecuador, 30.7% [30]; and Mexico, 22.6% [50].

Most people who presented with anxiety in our study were young This was also found in another study, where 59.8% of the participants were between 18 and 24 [4]. The different prevalence values reported may be due to several factors. Among them, we can mention the following: the use of different instruments in each study (STAI, STAI Y-2, and DASS-21) [30,44,48,50]; the timing of the studies (for example, in the first ten weeks of the pandemic [51]); and the context of each country, including the number of people infected with COVID-19 or the number of deaths from this disease, in addition to inaccurate or exaggerated information in the media [52], which caused anxiety about health status to become excessive [53].

The current pandemic has characteristics that may favor the appearance of higher levels of stress reaction, compared with other pandemics, such as its status as a new virus and the insidious course of the disease, which generates uncertainty about its management. This context explains the high prevalence of mental disorders found in this study and elsewhere [54]. Therefore, it is necessary to develop screening programs to diagnose anxiety and to provide early treatment.

### 4.2. Resilience 

We found that the median (interquartile range) resilience score was 24 points (IQR: 13–31). Similar results were found in a study conducted in the United States with the same measurement instrument, where a median of 29.5 was obtained. That score was associated with a higher use of adaptive coping behaviors [55]. is the United States score was slightly higher, compared with our findings; this was probably due to the sample (older adults), as it people aged 85 years or older demonstrate the same or even a greater resilience capacity than younger people [55]. Another study reported that adults are the most resilient group with respect to emotional regulation and problem-solving capacity [56].

### 4.3. Resilience and Mental Health Disorders

Resilience is one of the best predictors of an individual’s mental health status. It is moderately positively correlated with mental health [57]. Resilience was evaluated as a potentially protective factor during the COVID-19 pandemic, in regard to quality of life [58], PTSD, depression, anxiety [59], and well-being [60] in the general population. Resilience has been documented to help reduce PTSD symptoms [61]. It is also a protective factor, because the impact of a new-onset stressor is mitigated in people with greater resilience, before being exposed to the trauma [59]. 

However, in our study, we only found an association between resilience and PTSD due to the pandemic, which was only 1.0% higher. This suggested the existence of a spurious association. In a study of hospitalized patients with COVID-19, there was no evidence of difference between resilience and PTSD [62]. Another study conducted in the general population found a negative correlation between resilience and mental health outcomes [63]. This may have been due to the experience of the first wave, the knowledge acquired to avoid increases in contagion, and the need to overcome adversity. 

### 4.4. Factors Associated with PTSD

The pandemic is a global stressor and simultaneously affects several areas of people’s wellbeing, such as the financial, relational, and health domains. This stress can exacerbate feelings associated with PTSD symptoms [32]. In our study, we found several factors associated with PTSD, such as having had a family member who died from COVID-19, similar to what was reported in the United States, where bereaved people showed a higher risk of functional impairment if they had PTSD symptoms [64]. Our results could reflect that, in many cases, people saw their relatives die at home because of the lack of access to and the collapse of health services. This situation prevented them from being with their relatives at the time of hospitalization, due to the restrictions imposed and the insidious course of an unknown disease. 

Job loss was another positively associated factor, and this was similar to a systematic review that showed that people with no income, such as students, housewives, or unemployed people, were more susceptible to develop PTSD symptoms and stress [65]. Moreover, people with hypersomnia were more likely to develop PTSD. A similar result was reported in this study, where poor sleep quality correlated with higher levels of PTSD symptoms [66]. 

### 4.5. Factors Associated with Depression and Anxiety

Having had a family member who died from COVID-19, a job loss, or a feeling of food insecurity were found to be associated with anxiety and depression. Similar results were reported in studies conducted among relatives of deceased victims. These studies stated that people who are bereaved become vulnerable, due to the various psychological crises that affect their mental health, harboring two worrisome circumstances such as “emotional shock and fear of the future or uncertainty” [67,68]. Our result is supported by a study that evaluated an association between food security and mental health. It was found that the probability of developing anxiety and depression was 257.0% and 253.0% higher, respectively, in people who suffered from food insecurity, which was three times higher than the probability for people who had lost their jobs, where the risk was 32.0% and 27.0%, respectively [69].

In our study, hypersomnia was found to be associated with increased depression and anxiety. Previous studies identified that sleep disturbances were associated with higher levels of anxiety and depression [70,71,72,73]. However, a recent study showed that anxiety due to COVID-19 was correlated positively with severe insomnia [74]. This situation could be attributed to the excessive use of television, as well as to the constant use of social networks [75], as they are forms of distraction that are more greatly preferred after confinement due to restrictive measures imposed by the government. In addition, it has been found that loneliness, as a result of confinement, is associated with increased cortisol secretion when waking up, which explains its relationship with mental health disorders [76]. In this situation, the implementation of an intervention for sleep quality would reduce mental health disorders. 

Depression and having complied with containment measures, such as confinement and/or isolation and/or social distancing, turned out to be associated variables. This was similar to the findings of a systematic review that indicated that long periods of social distancing contributed to the occurrence of depressive symptoms [65]. These results were supported by a study in Saudi Arabia, where it was found that maintaining at least one meter of social distancing was significantly associated with lower scores on stress and anxiety, but not on depression [46].

Another factor associated with depression was having an incomplete higher education. This finding was similar to the result of a study in Spain, where being a student was a predictor of depressive symptoms because younger people are less mature and have fewer personal resources to face a crisis [77]. This situation is more pronounced in people with lower levels of education [65]. Inequalities in the financial sphere and in distress levels limit this age group in seeking timely care. The government should provide the necessary resources to reverse this situation. 

In addition, it was found that being a woman was associated with greater symptoms of depression and anxiety. A similar study showed that women have a worse reaction to confinement than men. This situation was more common in married women with a high educational level and low income [78]. This may be due to the fact that women have different personal and family responsibilities. Some research studies have shown that role pressure in the work and the home domains generates negative consequences for mental health [79]. A similar situation was found in the case of the presence of anxiety [80], which is associated with fear of COVID-19 infection and the worsening of the economy [81]. 

Resilience is known to be a protective factor in the face of fortuitous and/or stressful events, such as the period of confinement and other restrictions due to the COVID-19 pandemic. We know that having a coping strategy during the pandemic should help in controlling the development of mental disorders [81]. However, resilience did not show an association with depression or anxiety.

To sum up, several stressors have been identified due to confinement, such as prolonged time, fear of confinement, boredom, economic losses, and frustration [82]. There are other highly stressful events that could influence on the development of mental disorders, such as the role of being a parent [83], in which the parenting style has a relationship with children’s adjustment during childhood and later adult life [84,85]. Specifically, it has been shown that warmth has a beneficial role for children’s mental health and adjustment, while strictness seems to have no effect in reducing the risk of mental disorders [84,85,86,87]. Despite this, the factors identified in this study will serve as background information for other situations of such magnitude.

### 4.6. Relevance for Public Health

This study provides insight into resilience as a protective factor in an unanticipated context. The role of resilience has been considered previously, but its theoretical framework has remained complex and it is influenced by various factors, such as culture, religion, and social aspects. This study provides information that helps in understanding how the pandemic influenced a middle-income country that was greatly affected by the pandemic, compared with other countries.

### 4.7. Limitations and Strengths

In relation to the study’s limitations, the cross-sectional study design did not allow us to identify causal relationships between the study variables, but as a strength, validated instruments on PTSD, anxiety, depression, sleep quality, and resilience were used in our context. In addition, our study could have had selection bias due to a lack of representativeness, as the chosen sampling was of the non-probability snowball type, with the largest number of participants being university students (65.0%), a situation that reflects that the age and occupation groups in the study were not equitable. However, a strength of the study was that it was conducted in eight high-altitude cities. Moreover, it was a multicenter study, which covered remote regions of the center of Peru where the conditions were totally different. 

Another limitation is that the results cannot be extrapolated and measurement biases may occur because the questionnaires were self-administered; however, information was obtained from different groups with different occupations, which was a strength of the study. 

Finally, although measures of mental disorders were taken before the pandemic, it is possible that other variables could have influenced the development of these conditions. Therefore, it is possible that the results obtained were not entirely due to the pandemic. Nonetheless, the information provided here may support or contrast further hypotheses.

## 5. Conclusions

Our findings show that, during the pandemic, the prevalence of mental disorders was higher than in other non-pandemic contexts. Special attention should be paid to the factors that influence the development of mental health disorders to plan interventions and to be able to prevent their consequences. Furthermore, there should be mental health prevention and promotion programs, in addition to counseling to strength our community centers and obtain timely and rapid detection of mental health disorders. Our results provide information for the implementation of policies regarding mental health care of the general population and serve as background information in the event of an unforeseen future event. Furthermore, these results are of importance in medicine because the updated prevalence will allow the development of studies in other contexts, particularly in Latin America. 

## Figures and Tables

**Table 1:** Characteristics of the participants (*n* = 700).

Characteristics	*n* (%) or Median (Interquartile Range)
Age (years) *	23 (21–30)
**Sex**	
Female	394 (56.7)
Male	301 (43.3)
**Time at home**	
12 to 24 h	508 (72.6)
7 to 12 h	140 (20)
1 to 6 h	52 (7.4)
**Perception of health**	
Very good	85 (12.1)
Good	391 (55.8)
Fair	200 (28.6)
Bad	21 (3.0)
Very bad	3 (0.4)
**Level of education**	
Without formal education	1 (0.1)
Incomplete primary	3 (0.4)
Complete primary	4 (0.6)
Incomplete secondary	24 (3.4)
Complete secondary	139 (19.9)
Incomplete higher education	310 (44.3)
Complete higher education	164 (23.4)
Postgraduate	55 (7.9)
**Occupation**	
Housewife/retired	30 (4.3)
Public/private institutions employees	162 (23.1)
University/technical students	455 (65.0)
Others	18 (2.6)
**Religion**	
Catholic	447 (63.9)
Evangelical	91 (13.0)
Other	72 (10.3)
None	90 (12.9)
**Comorbidity history**	
No	595 (85.0)
Yes	105 (15.0)
**Previous comorbidity**	
None	596 (85.1)
Asthma	12 (1.7)
Diabetes mellitus	1 (0.1)
Arterial hypertension	10 (1.4)
Obesity	13 (1.9)
Others	64 (9.1)
**Self-report of a diagnosis of COVID-19**	
No	611 (87.3)
Yes	89 (12.7)
**Perception of the compliance with the contention measures**	
No	106 (15.1)
Yes	594 (84.9)
**Perception of the severity of the COVID-19 pandemic**	
Very serious	281 (40.1)
Serious	312 (44.6)
Neutral	68 (9.7)
Overrated	31 (4.4)
Really overrated	8 (1.1)
**Degree of confidence in the government to manage the pandemic**	
Much confidence	30 (4.3)
Some confidence	306 (43.7)
Neither trusts nor distrusts	172 (24.6)
Some distrust	117 (16.7)
Much distrust	75 (10.7)
**Previous mental disease**	
No	637 (91.0)
Yes	63 (9.0)
**Mental disorder in specific categories**	
None	637 (91.0)
Anxiety	17 (2.4)
Depression	14 (2.0)
Bipolar disorder	4 (0.6)
Obsessive Compulsive Disorder	8 (1.1)
Post-traumatic disorder	5 (0.7)
Others	15 (2.2)
**Family members with COVID-19**	
No	333 (47.6)
Yes	367 (52.4)
**Family member who died from COVID-19**	
No	557 (79.6)
Yes	143 (20.4)
**Search of mental health support**	
No	612 (87.4)
Yes	88 (12.6)
**Job loss due to the COVID-19 pandemic**	
Did not work	374 (53.4)
Did not lose their job	226 (32.2)
Lost their job	100 (14.3)
**Anxiety**	
No	191 (27.3)
Yes	509 (72.7)
**Anxiety degree**	
Absence	191 (27.3)
Mild	269 (38.4)
Moderate	152 (21.7)
Severe	88 (12.6)
**Depression**	
No	251 (35.9)
Yes	449 (64.1)
**Degree of depression**	
Minimal	251 (35.9)
Mild	209 (29.9)
Moderate	124 (17.7)
Moderate–severe	68 (9.7)
Severe	48 (6.9)
**Post-traumatic stress disorder**	
No	594 (84.8)
Yes	106 (15.1)
Resilience *	24 (13–31)
Insomnia *	17 (12–22)
Hypersomnia *	6 (4–8)
**Food insecurity**	
Food security	440 (62.9)
Mild food insecurity	123 (17.6)
Moderate food insecurity	72 (10.3)
Severe food insecurity	65 (9.3)

* Median (interquartile range). Some values may not add up to *n* = 700 due to missing data.

**Table 2:** Factors associated with post-traumatic stress, depression, and anxiety.

Variables	Post-Traumatic Stress Disorder			Depression			Anxiety		
	No	Yes	*p* *	No	Yes	*p* *	No	Yes	*p* *
	*n* (%)	*n* (%)		*n* (%)	*n* (%)		*n* (%)	*n* (%)	
Age **^,^†			**0.005**			**<0.001**			0.012
Young	427 (82.6)	90 (17.4)		161 (31.1)	356 (68.9)		128 (24.8)	389 (75.2)	
Adult	167 (91.3)	16 (8.7)		90 (49.1)	93 (50.8)		63 (34.4)	120 (65.6)	
**Sex**			0.993			**0.003**			**<0.001**
Female	335 (85.0)	59 (15.0)		123 (31.2)	271 (68.8)		82 (20.8)	312 (79.2)	
Male	256 (85.0)	45 (15.0)		127 (42.1)	174 (57.8)		108 (35.8)	193 (64.1)	
**Level of education**			**0.021**			**<0.001**			0.225
Not higher	151 (88.3)	20 (11.7)		52 (30.4)	119 (69.6)		45 (26.3)	126 (73.7)	
Incomplete higher education	250 (80.7)	60 (19.4)		98 (31.6)	212 (68.4)		77 (24.8)	233 (75.2)	
Complete higher/undergraduate	193 (88.1)	26 (11.9)		101 (46.1)	118 (53.9)		69 (31.5)	150 (68.5)	
**Occupation**			**0.004**			**<0.001**			0.057
No	399 (82.3)	86 (17.7)		149 (30.7)	336 (69.3)		122 (25.2)	363 (74.9)	
Yes	195 (90.7)	20 (9.3)		102 (47.4)	113 (52.6)		69 (32.1)	146 (67.9)	
**Religion**			0.091			0.441			0.383
No	71 (78.9)	19 (21.1)		29 (32.2)	61 (67.8)		28 (31.1)	62 (68.9)	
Yes	523 (85.7)	87 (14.3)		222 (36.4)	388 (63.6)		163 (26.7)	447 (73.3)	
**Comorbidity history**			**<0.001**			**<0.001**			0.006
No	517 (86.9)	78 (13.1)		231 (38.8)	364 (61.1)		174 (29.2)	421 (70.8)	
Yes	77 (73.3)	28 (26.7)		20 (19.1)	85 (81.0)		17 (16.2)	88 (83.8)	
**Self-report of diagnosis of COVID-19**			0.425			0.651			0.109
No	521 (85.3)	90 (14.7)		221 (36.2)	390 (63.8)		173 (28.3)	438 (71.1)	
Yes	73 (82.0)	16 (18.0)		30 (33.7)	59 (66.3)		18 (20.2)	71 (79.8)	
**Time at home**			0.051			0.065			0.036
12–24 h	422 (83.0)	86 (16.9)		169 (33.3)	339 (66.7)		127 (25.0)	381 (75.0)	
7–12 h	128 (91.4)	12 (8.6)		59 (42.1)	81 (57.9)		43 (30.7)	97 (69.3)	
1–6 h	44 (84.6)	8 (15.4)		23 (44.2)	29 (55.8)		21 (40.4)	31 (59.6)	
**Perception of health**			**<0.001**			**<0.001**			**<0.001**
Good/very good	425 (89.3)	51 (10.7)		210 (44.1)	266 (55.9)		157 (33.0)	319 (67.0)	
Fair	153 (76.5)	47 (23.5)		36 (18.0)	164 (82.0)		29 (14.5)	171 (85.5)	
Bad/very bad	16 (66.7)	8 (33.3)		5 (20.8)	19 (79.2)		5 (20.8)	19 (79.2)	
**Perception of the compliance with the contention measures**			0.546			0.028			0.489
No	92 (86.8)	14 (13.2)		28 (26.4)	78 (73.6)		26 (24.5)	80 (75.5)	
Yes	502 (84.5)	92 (15.5)		223 (37.5)	371 (62.5)		165 (27.8)	429 (72.2)	
**Perception of the severity of the COVID-19 pandemic**			0.895			0.336			0.435
Serious/very serious	503 (84.8)	90 (15.2)		211 (35.6)	382 (64.4)		160 (27.0)	433 (73.0)	
Neutral	57 (83.8)	11 (16.2)		22 (32.4)	46 (67.7)		17 (25.0)	51 (75.0)	
Overrated/really overrated	34 (87.2)	5 (12.8)		18 (46.1)	21 (53.9)		14 (35.9)	25 (64.1)	
**Previous mental disease**			**<0.001**			**<0.001**			**<0.001**
No	560 (87.9)	77 (12.1)		247 (38.8)	390 (61.2)		187 (29.4)	450 (70.6)	
Yes	34 (54.0)	29 (46.0)		4 (6.4)	59 (93.7)		4 (6.4)	59 (93.7)	
**Family members with COVID-19**			0.252			0.014			0.010
No	288 (86.5)	45 (13.5)		135 (40.5)	198 (59.5)		106 (31.8)	227 (68.2)	
Yes	306 (83.4)	61 (16.6)		116 (31.6)	251 (68.4)		85 (23.2)	282 (76.8)	
**Family member who died from COVID-19**			**0.015**			**<0.001**			0.006
No	482 (86.5)	75 (13.5)		218 (39.1)	339 (60.9)		165 (29.6)	392 (70.4)	
Yes	112 (78.3)	31 (21.7)		33 (23.1)	110 (76.9)		26 (18.2)	117 (81.8)	
**Search of mental health support**			**<0.001**			0.023			0.010
No	528 (86.3)	84 (13.7)		229 (37.4)	383 (62.6)		177 (28.9)	435 (71.1)	
Yes	66 (75.0)	22 (25.0)		22 (25.0)	66 (75.0)		14 (15.9)	74 (84.1)	
**Lost his/her job due to the COVID-19 pandemic**			**<0.001**			**<0.001**			0.168
Did not work	319 (85.2)	55 (14.7)		116 (31.0)	258 (69.0)		93 (24.9)	281 (75.1)	
Did not lose his/her job	202 (89.3)	24 (10.6)		107 (47.3)	119 (52.7)		72 (31.9)	154 (68.1)	
Lost his/her job	73 (73.0)	27 (27.0)		28 (28.0)	72 (72.0)		26 (26.0)	74 (74.0)	
**Food insecurity**			**<0.001**			**<0.001**			**0.002**
Food security	392 (89.1)	48 (10.9)		186 (42.3)	254 (57.7)		142 (32.2)	298 (67.7)	
Mild food insecurity	108 (87.8)	15 (12.2)		34 (27.6)	89 (72.4)		23 (18.7)	100 (81.3)	
Moderate food insecurity	51 (70.8)	21 (29.1)		21 (29.2)	51 (70.8)		15 (20.8)	57 (79.2)	
Severe food insecurity	43 (66.1)	22 (33.8)		10 (15.4)	55 (84.6)		11 (16.9)	54 (83.1)	
Insomnia **^,^†	16 (12–20)	24 (20–29)	**<0.001**	12 (10–15)	20 (16–24)	**<0.001**	12 (10–15)	19 (15–24)	**<0.001**
Hypersomnia **^,^†	6 (4–7)	10 (7–12)	**<0.001**	4 (3–6)	7 (5–9)	**<0.001**	4 (3–6)	7 (5–9)	**<0.001**
Resilience **^,^†	25 (10–31)	24 (19–29)	0.595	26 (4–34)	24 (16–30)	0.607	26 (4–35)	24 (15–30)	0. 210

* *p* value estimated with the chi-squared test. ** *p* value estimated with the Mann–Whitney U test. † Median (interquartile range). The highlighted data represent the statistical significance found.

**Table 3:** Resilience and other factors associated with PTSD in the simple and multiple regression analysis.

Characteristics	Post-Traumatic Stress Disorder					
	Simple Regression			Multiple Regression		
	PR	95% CI	*p* *	PR	95% CI	*p* *
**Sex**						
Male	Ref.			Ref.		
Female	1.00	0.66–1.51	0.994	0.85	0.60–1.19	0.339
**Adults**						
No	Ref.			Ref.		
Yes	0.50	0.29–0.86	0.013	0.87	0.57–1.33	0.518
**Level of education**						
Other	Ref.			Ref.		
Incomplete higher/ongoing studies	1.65	0.90–3.06	0.108	1.14	0.68–1.92	0.618
Complete higher/undergraduate	1.02	0.40–2.58	0.975	1.40	0.60–3.26	0.439
**Currently working**						
No	Ref.			Ref.		
Yes	0.52	0.27–1.04	0.064	0.53	0.28–0.99	0.049
**Religion**						
No	Ref.			Ref.		
Yes	0.68	0.45–1.01	0.057	0.96	0.63–1.43	0.843
**Previous pathology**						
No	Ref.			Ref.		
Yes	2.03	1.55–2.67	<0.001	1.19	0.99–1.42	0.070
**Self-report of diagnosis of COVID-19**						
No	Ref.			Ref.		
Yes	1.22	0.81–1.83	0.336	1.20	0.76–1.90	0.433
**Time at home**						
12–24 h	Ref.			Ref.		
7–12 h	0.51	0.27–0.94	0.032	0.92	0.46–1.85	0.820
1–6 h	0.91	0.52–1.60	0.739	1.64	0.95–2.84	0.076
**Perception of health**						
Bad/very bad	Ref.			Ref.		
Fair	0.71	0.36–1.38	0.306	1.66	0.92–2.98	0.094
Good/very good	0.32	0.18–0.57	<0.001	1.23	0.70–2.18	0.468
**Perception of the compliance with the contention measures**						
Yes	Ref.			Ref.		
No	0.85	0.48–1.50	0.582	0.76	0.41–1.41	0.382
**Perception of the severity of the COVID-19 pandemic**						
Neutral	Ref.			Ref.		
Serious/very serious	0.94	0.53–1.66	0.827	0.89	0.68–1.16	0.395
Overrated/really overrated	0.79	0.29–2.12	0.644	1.09	0.34–3.41	0.889
**Previous mental disease history**						
No	Ref.			Ref.		
Yes	3.81	2.44–5.95	<0.001	1.88	0.93–3.79	0.078
**Family member with COVID-19**						
No	Ref.			Ref.		
Yes	1.23	0.80–1.89	0.340	0.92	0.68–1.23	0.562
**Family member who died from COVID-19**						
No	Ref.			Ref.		
Yes	1.61	1.28–2.03	<0.001	1.34	1.02–1.74	0.033
**Search of mental health support**						
No	Ref.			Ref.		
Yes	1.82	1.33–2.49	<0.001	1.16	0.99–1.35	0.055
**Lost his/her job due to the COVID-19 pandemic**						
Did not work	Ref.			Ref.		
Did not lose his/her job	0.72	0.46–1.13	0.153	1.44	1.10–1.87	0.007
Lost his/her job	1.84	1.37–2.47	<0.001	2.21	1.37–3.56	0.001
**Food insecurity**						
Food security	Ref.			Ref.		
Mild food insecurity	1.12	0.70–1.63	0.562	1.04	0.71–1.53	0.824
Moderate food insecurity	2.67	1.98–3.60	<0.001	1.37	1.05–1.78	0.022
Severe food insecurity	3.10	1.79–5.39	<0.001	1.49	0.92–2.41	0.103
**Insomnia**	1.16	1.14–1.17	<0.001			
**Hypersomnia**	1.32	1.24–1.40	<0.001	1.27	1.19–1.35	<0.001
**Resilience**	1.01	1.00–1.03	0.032	1.01	1.00–1.03	0.032

* *p* values obtained through the Generalized Linear Model (GLM), Poisson family, log link function, robust variance, and clustering by place of residence.

**Table 4:** Resilience and other factors associated with depression and anxiety in the simple and multiple regression analysis.

Characteristics	Depression						Anxiety					
	Simple Regression			Multiple Regression			Simple Regression			Multiple Regression		
	PR	95% CI	*p* *	PR	95% CI	*p* *	PR	95% CI	*p* *	PR	95% CI	*p* *
**Gender**												
Male	Ref.			Ref.			Ref.			Ref.		
Female	1.19	1.07–1.32	0.001	1.08	0.98–1.20	0.135	1.24	1.17–1.31	<0.001	1.14	1.03–1.28	**0.011**
**Adults**												
No	Ref.			Ref.			Ref.			Ref.		
Yes	0.74	0.57–0.96	0.023	0.90	0.68–1.00	0.467	0.87	0.73–1.04	0.126	0.93	0.79–1.09	0.347
**Level of education**												
Other	Ref.			Ref.			Ref.			Ref.		
Incomplete higher/ongoing studies	0.98	0.88–1.09	0.760	0.91	0.84–0.98	**0.011**	1.02	0.95–1.10	0.608	0.97	0.91–1.03	0.307
Complete higher/undergraduate	0.77	0.66–0.90	0.001	0.90	0.78–1.06	0.221	0.93	0.89–0.98	0.003	0.97	0.90–1.07	0.642
**Currently working**												
No	Ref.			Ref.			Ref.			Ref.		
Yes	0.76	0.64–0.90	0.001	0.95	0.86–1.04	0.273	0.91	0.79–1.04	0.152	1.05	0.91–1.21	0.493
**Religion**												
No	Ref.			Ref.			Ref.			Ref.		
Yes	0.94	0.79–1.12	0.476	1.03	0.88–1.21	0.711	1.06	0.96–1.18	0.228	1.11	0.97–1.26	0.123
**Previous pathology**												
No	Ref.			Ref.			Ref.			Ref.		
Yes	1.32	1.25–1.41	<0.001	1.09	0.94–1.26	0.264	1.18	1.08–1.29	<0.001	1.06	0.93–1.21	0.417
**Self-report of diagnosis of COVID-19**												
No	Ref.			Ref.			Ref.			Ref.		
Yes	1.04	0.80–1.35	0.775	0.99	0.80–1.22	0.915	1.11	1.01–1.22	0.022	1.06	0.97–1.15	0.209
**Time at home**												
12–24 hrs.	Ref.			Ref.			Ref.			Ref.		
7–12 hrs.	0.87	0.70–1.08	0.205	1.02	0.88–1.18	0.810	0.92	0.82–1.03	0.162	0.98	0.88–1.09	0.696
1–6 hrs.	0.84	0.61–1.15	0.265	0.90	0.72–1.16	0.437	0.79	0.57–1.10	0.166	0.79	0.62–1.02	0.070
**Perception of health**												
Bad/very bad	Ref.			Ref.			Ref.			Ref.		
Fair	1.04	0.82–1.31	0.772	1.22	0.96–1.56	0.102	2.01	1.69–2.41	<0.001	1.16	0.97–1.38	0.102
Good/very good	0.71	0.58–0.85	<0.001	1.01	0.82–1.25	0.922	1.70	1.32–2.21	<0.001	1.04	0.92–1.19	0.506
**Perception of the compliance with the contention measures**												
Yes	Ref.			Ref.			Ref.			Ref.		
No	1.18	1.00–1.38	0.042	1.20	1.08–1.34	**0.001**	1.04	0.92–1.19	0.501	1.07	0.98–1.16	0.117
**Perception of the severity of the COVID-19 pandemic**												
Neutral	Ref.			Ref.			Ref.			Ref.		
Serious/very serious	0.95	0.81–1.12	0.548	0.93	0.81–1.07	0.323	0.97	0.87–1.09	0.639	0.93	0.82–1.05	0.246
Overrated/really overrated	0.79	0.61–1.03	0.084	0.87	0.71–1.09	0.239	0.85	0.73–0.99	0.050	0.93	0.79–1.09	0.365
**Previous mental disease history**												
No	Ref.			Ref.			Ref.			Ref.		
Yes	1.53	1.38–1.69	<0.001	1.15	0.99–1.33	0.054	1.33	1.24–1.41	<0.001	1.09	0.97–1.22	0.135
**Family member with COVID-19**												
No	Ref.			Ref.			Ref.			Ref.		
Yes	1.15	1.03–1.29	0.015	0.99	0.86–1.14	0.934	1.13	1.05–1.21	0.001	1.02	0.96–1.09	0.535
**Family member who died from COVID-19**												
No	Ref.			Ref.			Ref.			Ref.		
Yes	1.26	1.12–1.43	<0.001	1.19	1.08–1.31	**0.001**	1.16	1.09–1.24	<0.001	1.10	1.02–1.19	**0.011**
**Search of mental health support**												
No	Ref.			Ref.			Ref.			Ref.		
Yes	1.19	1.05–1.37	0.007	1.00	0.93–1.09	0.859	1.18	1.14–1.22	<0.001	1.05	0.99–1.11	0.134
**Lost his/her job due to the COVID-19 pandemic**												
Did not work	Ref.			Ref.			Ref.			Ref.		
Did not lose his/her job	0.76	0.63–0.93	0.006	0.99	0.83–1.19	0.983	0.91	0.81–1.01	0.087	1.06	0.97–1.17	0.197
Lost his/her job	1.04	0.91–1.19	0.535	1.10	1.03–1.29	**0.007**	0.98	0.85–1.14	0.838	1.01	0.85–1.19	0.904
**Food insecurity**												
Food security	Ref.			Ref.			Ref.			Ref.		
Mild food insecurity	1.25	1.21–1.29	<0.001	1.19	1.10–1.29	**<0.001**	1.20	1.11–1.29	<0.001	1.17	1.06–1.29	**0.002**
Moderate food insecurity	1.23	0.99–1.51	0.055	1.02	0.83–1.25	0.835	1.17	1.00–1.36	0.042	1.06	0.82–1.36	0.674
Severe food insecurity	1.47	1.39–1.54	<0.001	1.17	1.10–1.25	**<0.001**	1.23	1.14–1.31	<0.001	1.07	0.92–1.26	0.380
**Insomnia**	1.06	1.05–1.07	<0.001				1.04	1.03–1.05	<0.001			
**Hypersomnia**	1.11	1.09–1.12	<0.001	1.09	1.07–1.11	**<0.001**	1.08	1.07–1.09	<0.001	1.07	1.05–1.08	**<0.001**
**Resilience**	1.00	0.99–1.01	0.356	1.00	0.99–1.01	0.200	1.00	0.99–1.00	0.697	1.00	0.99–1.00	0.535

* *p* values obtained through the Generalized Linear Model (GLM), Poisson family, log link function, robust variance, and clustering by place of residence. The highlighted data represent the statistical significance found.

## Data Availability

Not applicable.
